# Towards a Better Understanding of Cognitive Deficits in Absence Epilepsy: a Systematic Review and Meta-Analysis

**DOI:** 10.1007/s11065-019-09419-2

**Published:** 2019-11-27

**Authors:** Eric L. A. Fonseca Wald, Jos G. M. Hendriksen, Gerald S. Drenthen, Sander M. J. V. Kuijk, Albert P. Aldenkamp, Johan S. H. Vles, R. Jeroen Vermeulen, Mariette H. J. A. Debeij-van Hall, Sylvia Klinkenberg

**Affiliations:** 1 Department of Neurology, Maastricht University Medical Center+, 6202 AZ Maastricht, The Netherlands; 2 Epilepsy Center Kempenhaeghe, Heeze, The Netherlands; 3 School for Mental Health and Neuroscience, Maastricht University, Maastricht, The Netherlands; 4 Department of Radiology and Nuclear Medicine, Maastricht University Medical Center+, Maastricht, The Netherlands; 5 Department of KEMTA, Maastricht University Medical Center+, Maastricht, The Netherlands; 6 Department of Electrical Engineering, Eindhoven University of Technology, Eindhoven, The Netherlands

**Keywords:** Absence epilepsy, Cognition, Meta-analysis, Systematic review

## Abstract

Cognition in absence epilepsy (AE) is generally considered undisturbed. However, reports on cognitive deficits in AE in recent years have suggested otherwise. This review systematically assesses current literature on cognitive performance in children with AE. A systematic literature search was performed in Pubmed, Embase, Cochrane and Web of Science. All studies reporting on cognitive performance in children with AE were considered. In total 33 studies were eligible for inclusion. Neuropsychological tests were classified into the following domains: intelligence; executive function; attention; language; motor & sensory-perceptual examinations; visuoperceptual/visuospatial/visuoconstructional function; memory and learning; achievement. Random-effect meta-analyses were conducted by estimating the pooled mean and/or pooling the mean difference in case-control studies. Full-scale IQ in children with AE was estimated at 96.78 (95%CI:94.46–99.10) across all available studies and in case-control studies IQ was on average 8.03 (95%CI:-10.45- -5.61) lower. Verbal IQ was estimated at 97.98 (95%CI:95.80–100.16) for all studies and 9.01 (95%CI:12.11- -5.90) points lower in case-control studies. Performance IQ was estimated at 97.23 (93.24–101.22) for all available studies and 5.32 (95%CI:-8.27–2.36) points lower in case-control studies. Lower performance was most often reported in executive function (cognitive flexibility, planning, and verbal fluency) and attention (sustained, selective and divided attention). Reports on school difficulties, neurodevelopmental problems, and attentional problems were high. In conclusion, in contrast to common beliefs, lower than average neurocognitive performance was noted in multiple cognitive domains, which may influence academic and psychosocial development.

**Electronic supplementary material:**

The online version of this article (10.1007/s11065-019-09419-2) contains supplementary material, which is available to authorized users.

## Introduction

Typical absence seizures as occurring in childhood absence epilepsy (CAE) and juvenile absence epilepsy (JAE) are characterized by demarcated brief episodes of unconsciousness with generalized ~3-Hz spike-and-wave complexes, visible on an electroencephalogram (EEG), in otherwise healthy children (Guerrini, 2006; Myers & Fecske, 2016; Panayiotopoulos, 2001; Tenney & Glauser, 2013). A clear delineation of the clinical spectrum between CAE and JAE is challenging (Hughes, 2009; Tenney & Glauser, 2013; Trinka et al., 2004). CAE occurs mostly between 4 and 10 years of age whereas JAE occurs between 10 and 17 years of age. Furthermore, JAE is characterized by less frequent absence seizures, a higher incidence of tonic-clonic seizures and a higher drug dependency during adulthood.

Despite absence epilepsy (AE) being defined a “benign” disorder, a high rate of initial treatment failure, associated therapeutic side effects, development of generalized tonic-clonic seizures and psychosocial co-morbidities in more recent reports emphasize its real burden (Bouma, Westendorp, van Dijk, Peters, & Brouwer, 1996; Caplan et al., 2008; Cnaan et al., 2017; Glauser et al., 2010; IJff et al., 2016; Loughman, Bendrups, & D'Souza, 2016; Masur et al., 2013).

Starting from early descriptions, cognitive performance in AE has been considered to be normal (Adie, 1924; Currier, Kooi, & Saidman, 1963). However, in a large randomized clinical trial intelligence was lower than normal values, but still within normal range (Masur et al., 2013). Nevertheless, 36% of drug-naïve patients presented with attentional deficits. Moreover, subsequent allocation to monotherapy with Valproate was associated with more attentional dysfunction, than allocation to monotherapy with either Ethosuximide or Lamotrigine (Cnaan et al., 2017; Glauser et al., 2013; Glauser et al., 2010). Nevertheless, attentional deficits persisted independent of the allocated anti-epileptic drug treatment or seizure control after 1-year of follow-up (Cnaan et al., 2017; Glauser et al., 2013; Masur et al., 2013). These are intriguing findings, as apart from therapeutic side effects and (inter)ictal activity other underlying mechanisms may also affect cognition in AE (Aldenkamp & Arends, 2004; Jafarian et al., 2015; Lenck-Santini & Scott, 2015; Nicolai et al., 2012).

The number of studies reporting on cognitive performance in AE has been growing steadily and warrants a formal review (Berg, Caplan, & Hesdorffer, 2011; Berg, Levy, Testa, & Blumenfeld, 2014; Caplan et al., 2008; Cerminara et al., 2013; Cheng et al., 2017; Conant, Wilfong, Inglese, & Schwarte, 2010; Conde-Guzon & Cancho-Candela, 2012; Covanis, Skiadas, Loli, Lada, & Theodorou, 1992; D’Agati, Cerminara, Casarelli, Pitzianti, & Curatolo, 2012; Fastenau et al., 2009; Franzoni et al., 2015; Gencpinar et al., 2016; Guerrini et al., 2015; Henkin et al., 2003; Henkin et al., 2005; Kernan et al., 2012; Levav et al., 2002; Lopes, Monteiro, Fonseca, Robalo, & Simoes, 2014; Lopes et al., 2013; Masur et al., 2013; Mostafa, Talaat, Shalaby, El-Fayoumy, & Labib, 2014; Nolan et al., 2004; Oostrom et al., 2003; Pavone et al., 2001; Schraegle, Nussbaum, & Stefanatos, 2016; Shinnar et al., 2017; Sinclair & Unwala, 2007; Siren et al., 2007; Talero-Gutierrez, Sanchez-Torres, & Velez-van-Meerbeke, 2015; Urena-Hornos et al., 2004; Vanasse, Beland, Carmant, & Lassonde, 2005; Vega et al., 2010; Verrotti et al., 2011; Wirrell, Camfield, Camfield, Gordon, & Dooley, 1996). A previous meta-analysis in idiopathic generalized epilepsy found significant impairments in general cognitive ability and across a wide variety of other cognitive domains (Loughman, Bowden, & D'Souza, 2014). Sub-analyses were available for CAE, however, only four studies were included. Therefore, our review is aimed specifically to address all studies on cognitive performance in AE. Although, the different syndromes in idiopathic generalized epilepsy may constitute a biological continuum, and therefore resemble in their neuropsychological profile, specific cognitive deficits may still exist. Knowledge on cognitive deficits in AE may help clinician’s establish better neuropsychological batteries and direct precautionary measures, as cognitive deficits may go unnoticed by their surroundings (Masur et al., 2013). Therefore, this review systematically assesses current literature on cognitive performance in children with AE.

## Methods

This review adheres to the Preferred Reporting Items for Systematic Reviews and Meta-analysis guidelines (PRISMA) (Moher, Liberati, Tetzlaff, & Altman, 2009). The methods and procedures for this review are available in the paper with additional information provided in the supplemental materials. The review was not registered prior to conducting this review.

### Selection Eligibility

Selection criteria were defined according to PICOS: Participants = Children with AE, either defined as 3–4 Hz spike-wave complexes or a syndromic classification of CAE and/or JAE; Intervention/diagnostic = neuropsychological tests (batteries) or reports on school performance; Comparison = normative values (for example reports using standardized scores as these represents scores relative to the normative sample of the test) or a control group; Outcome = neuropsychological function based reported as a median/average score or prevalence of impairment based on cut-off values or a direct comparison of test scores with a control group; Design = Observational studies (cohort studies/case-control studies) or clinical trials (depending of the design of the clinical trial these data are also regarded as observational data, for example baseline neuropsychological results).

### Systematic Literature Search

The search strategy consisted of indexed terms and free text words on absence epilepsy in combination with terms on observational research and clinical trials (the search in Pubmed is provided as a Supplemental file). The following electronic databases were searched: Pubmed (until 29-09-2017), EMBASE (until 23-03-2017), Cochrane (until 05-04-2017) and Web of Science (all databases) (until 06-04-2017). Furthermore, references of included articles were hand searched to find additional relevant articles. In case multiple publications were available on the same study sample or if reports reported overlapping results, only the most recent data were used. Only studies written in English, Dutch or Spanish were considered. Firstly, titles and/or abstracts identified by the search were first screened by EFW to remove any unrelated hits. Secondly, the remaining abstracts were screened by EFW and another author (GSD or SK or MD or JH) based on the predefined selection criteria. The second researcher was blinded for journal, authors, title, date of publication and publication language. Any discrepancies were resolved by consensus or by screening the full-text subsequently. Eligibility assessment of full-text articles was performed by EFW and one of the neurologists (MD or SK). Any discrepancies were resolved by consensus or by consulting a third author.

### Data Extraction

The following data were extracted: year of publication, country, study design, inclusion/exclusion criteria, number of included patients, number of patients per cognitive assessment, age at onset, age at the time of the study, anti-epileptic drug use and neuropsychological tests results.

Neuropsychological test results were classified to cognitive domains according to Baron and secondly according to the authors of the study or consensus within our team (Baron, 2004). We distinguished the following cognitive domains: intelligence; executive function; attention, language; motor and sensory-perceptual examinations; visuoperceptual/visuospatial/visuoconstructional function; learning and memory. Additionally, we included results on: achievement tests, parent/teacher (by proxy) reports on attention or attentional deficiency disorders; reported prevalence’s of school difficulties; neuropsychological and/or neurodevelopmental problems.

### Risk of Bias in Individual Studies

Risk of bias was evaluated using a modified version of the Newcastle Ottawa Scale made suitable for this review (available in the Supplementary Materials). Two authors (EFW & SK) independently rated each study, and any disagreement was resolved by consensus. For the interpretation of the total scores we used the following cut-off values as used previously in a systematic review (Marengoni et al., 2018). Scores >7 were considered a low risk of bias; 5 to 7, a moderate risk; and < 5, a high risk. For the meta-analysis sensitivity analyses were carried out by excluding studies with a score lower than seven.

### Meta-Analyses

Statistical analyses were conducted in R using the “meta” package (version 4.8–2) and “metaphor” package (version 1.9–9) (Schwarzer, 2007; Viechtbauer, 2010). Single-arm meta-analyses (a weighted pooling of the reported means including studies without a control group) were performed by estimating the weighted mean using a random effect model. For this, we used the reported mean and calculated the standard error (SE) for each study. In case a z-score with a 95% confidence interval was reported we calculated the standard score and its standard deviation. The estimated mean was considered significantly different from normal if the 95% confidence interval did not include the normative mean of the neuropsychological test.

In addition, a random-effects meta-analysis on the mean difference between cases and controls was performed. Studies that did not report usable data for pooling of results were discarded for the meta-analyses but were included in this systematic review. Pooling of results was conducted per neuropsychological test if methodology and reporting of the results allowed a direct comparison between studies. *P* values of ≤0.05 were considered to infer statistical significance.

The presence of small study effect and/or publication bias was assessed by visually inspecting funnel plots for asymmetry. In case of potential outliers, a sensitivity analysis was performed by recalculating the effect size after removal of these studies. Tau-squared (*T*^*2*^*)* was used to estimate the true variance of the true effect sizes (Borenstein, Higgins, Hedges, & Rothstein, 2017). In addition, I^2^ is reported for descriptive purposes. Due to the limited amount of studies in the meta-analyses we were not able to perform subsequent meta-regressions to examine the impact of moderator variables (Shuster, 2011; Thompson & Higgins, 2002).

## Results

### Selection of Studies

**Fig. 1:**
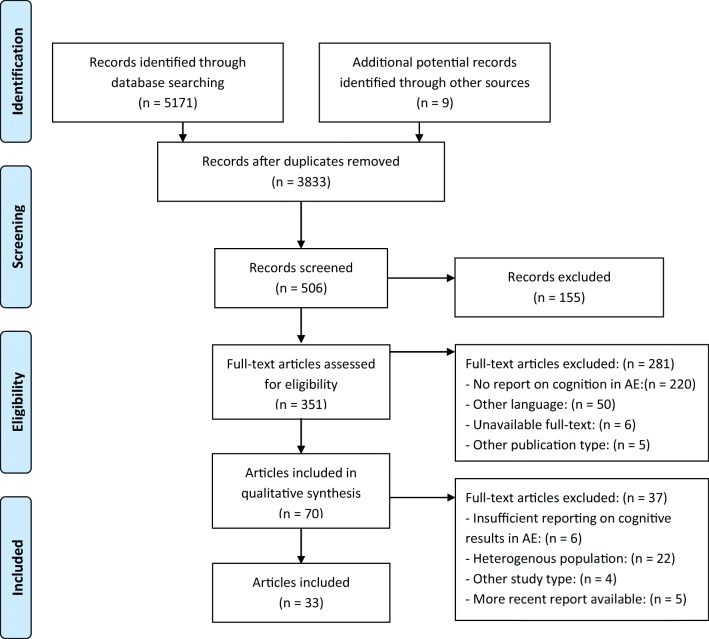
Flowchart (adapted from Moher et al. (2009))

The study selection process is depicted in Fig. 1. A total of 3833 individual articles across all electronic databases were screened. Additionally, nine references from selected articles were screened for eligibility. A total of 506 abstracts were selected for further reading, after which 351 were selected for full-text screening. In the end, 33 articles were included in this systematic review.

### Study Characteristics

Study characteristics of the 33 included articles are listed in Table 1. Eight articles reported on overlapping cohorts, but were included as each article provided complementary data in that cohort (Berg et al., 2011; Berg et al., 2014; Henkin et al., 2003; Henkin et al., 2005; Lopes et al., 2014; Lopes et al., 2013; Masur et al., 2013; Shinnar et al., 2017). In total, data were available for 29 samples. In total, 17 cohorts reported only on CAE (Berg et al., 2011; Berg et al., 2014; Caplan et al., 2008; Cerminara et al., 2013; Cheng et al., 2017; Conant et al., 2010; D’Agati et al., 2012; Gencpinar et al., 2016; Guerrini et al., 2015; Kernan et al., 2012; Levav et al., 2002; Lopes et al., 2014; Lopes et al., 2013; Masur et al., 2013; Mostafa et al., 2014; Nolan et al., 2004; Oostrom et al., 2003; Schraegle et al., 2016; Shinnar et al., 2017; Siren et al., 2007; Talero-Gutierrez et al., 2015; Vega et al., 2010; Wirrell et al., 1996) and in 12 cohorts reports were available in children with AE or a combination of CAE with JAE (Conde-Guzon & Cancho-Candela, 2012; Covanis et al., 1992; Fastenau et al., 2009; Franzoni et al., 2015; Henkin et al., 2003; Henkin et al., 2005; Oostrom et al., 2003; Pavone et al., 2001; Sinclair & Unwala, 2007; Siren et al., 2007; Talero-Gutierrez et al., 2015; Urena-Hornos et al., 2004; Vanasse et al., 2005; Verrotti et al., 2011). No studies were available reporting on JAE exclusively. Study sample sizes varied from 10 to 446, with a mean of 54 participants. The time of cognitive assessment differed from early assessment close to the time of diagnosis to years after diagnosis. Overall Valproic Acid (VPA) seems the most used anti-epileptic drug in the studies. However, some studies only reported the amount of anti-epileptic drug (AED) use (monotherapy, polytherapy) without stating the number of patients on particular AEDs.

**Table 1:** Study characteristics of included studies

Reference	Study design	Population	N	Age at onset (SD) in years	Age in study (SD) in years	AED use	Risk of Bias
Berg et al., 2011, 2014	Prospective	CAE	51–59	ESM 5.9 (1.8); VPA 6.4 (1.8)	NA	ESM 69%, VPA 31%	Low
Caplan et al., 2008	Case-control	CAE	69	6.15 (2.52)	9.64 (2.49)	No AED 12%, Monotherapy 76%- > VPA 51%, ESM 35%, Other 14%, Polytherapy 13%	Low
Cerminara et al., 2013	Case-control	CAE	24	NA	NA	VPA 18, LEV 4, LTG 1, VPA + LTG 1	Moderate
Cheng et al., 2017	Case-control	CAE	43	≤5: *n* = 14; >5 29	9.5 (2.6)	No AED 7%, Monotherapy 72%, Duotherapy 21%	Low
Conant et al., 2010	Case-control	CAE	16	4.5–8	8.0 (1.3)	No AED 6, VPA 10, VPA + ESM 1	Low
Conde-Guzon & Cancho-Candela, 2012	Case-control	AE	34	5.1 (1.2)	8.7 (1.26)	100% VPA	Low
Covanis et al., 1992	Retrospective	AE	124	2.5–13.5	NA	‡	Moderate
D’Agati et al., 2012	Case-control	CAE	15	8.8 (1.74)	11.46 (2.23)	VPA 100%	Moderate
Fastenau et al., 2009	Prospective	AE	38	6–14	NA	NA	Low
Franzoni et al., 2015	Retrospective	AE drug-resistant	92	5.33	3y minimal follow-up time	Polytherapy 100%, >3AED’s 47%, >5 AED’s 53%	Low
Gencpinar et al., 2016	Case-control	CAE	69	3–6; n = 14, ≥7 *n* = 5	12.22 (2.46)	Monotherapy 73.7%, Duotherapy 26.3%, Tripletherapy 26.3%	Moderate
Guerrini et al., 2015	Case-Control	CAE	82	6.28 (2.5)	9.7 (1.78)	NA	Low
Henkin et al., 2003, 2005	Case-control	AE	12	7.2	14.4 (1.83)	VPA 100%	Moderate
Kernan et al., 2012	Case-control	CAE	31	6 (2)	9 (2)	No AED 6%, Monotherapy 68%, Polytherapy 26%	Low
Levav et al., 2002	Case-control	CAE	28	5.6 (2–16)	14.0 (10.5)	No AED 18, Monotherapy 7, Polytherapy 3	Low
Lopes et al., 2013, 2014	Case-control	CAE	30	6.83 (2.32)	9.93 (2.54)	No AED 13%, Monotherapy 73%, Duotherapy 13%	Low
Masur et al., 2013; Shinnar et al., 2017	RCT	CAE	446	≥6 years of age *n* = 336 <6 years of age *n* = 110	Pre-treatment analysis (=max. 1 week after start AED)	After randomization: ESM 35%, LTM 33%, VPA 32%	Low
Mostafa et al., 2014	Cross-sectional	CAE	10	8.4 (1.9)	13 (4.1)	VPA 80%, LTG 20%	Low
Nolan et al., 2004	Prospective	CAE	13	5.5 (2.0)	9.5 (2.3)	Monotherapy 48%, Polytherapy 62%	Moderate
Oostrom et al., 2003	Prospective	CAE or JAE	10	NA	9.2 (1.9)	100% AED not specified	Moderate
Pavone et al., 2001	Case-control	AE	16	5.3	9.2 (3)	ESM 2, VPA 11, ESM + VPA 3	Low
Schraegle et al., 2016	Cross-sectional	CAE	30	4.83 (1.89)	11.1 (2.95)	Monotherapy 16%, Polytherapy 46.7%	Low
Sinclair & Unwala, 2007	Retrospective	AE	80	7.5 (2.7)	13.9 (3.2)	NA	Moderate
Siren et al., 2007	Case-control	CAE 9, JAE 1	10	3.0–11.8	8.2, 5.5–14.5	VPA 4, ESM 5, VPA+ESM 1	Low
Urena-Hornos et al., 2004	Retrospective	AE	49	7.93	10 days-13 years follow up	Monotherapy VPA 78%‡	Moderate
Vanasse et al., 2005	Case-control	AE	10	5.17 (2.26)	10.13 (1.69)	Monotherapy 5, Polytherapy 5	Moderate
Vega et al., 2010	Case-control	CAE	38	6.9 (2.8)	10.5 (2.3)	No AED 13.2%, Monotherapy 63.2%, Duotherapy 21.1%Polytherapy 2.6%	Low
Verrotti et al., 2011	Retrospective	AE before age of 3	40	2.2 (0.59)	8.19	No AED 2, VPA 26, LTG 1, LEV 1, ESM 3, VPA- > CLB 1, VPA->ESM 2, VPA- > LEV 1, VPA- > LTG 1, VPA->ESM 1, VPA- > CLB 1	Low
Wirrell et al., 1996	Retrospective	CAE	60	5.7 (2.8)	20.4 (4.2)	No AED 47, VPA 9, VPA + LTG 1, LTG 1 VPA+ESM 1, ESM + LTM + CBM 1	Moderate

a May contain overlapping patients, however reports on neuropsychological test results did not overlap ‡ Refer to original paper

AE, Absence Epilepsy; AED, Anti-Epileptic Drug; CAE, Childhood Absence Epilepsy; CBM, Carbamazepine; CLB, Clobazam; ESM, Ethosuximide; JAE, Juvenile Absence Epilepsy; LEV, Levetiracetam; LTG, Lamotrigine; N, number of patients; NA, Not available; RCT, Randomized Clinical Trial; SD, Standard Deviation; VPA, Valproic Acid

Based on our assessment for the risk of bias eighteen studies had a low risk of bias and eleven studies had a moderate risk of bias. No studies were classified to have a high risk of bias. The highest risk of bias was due to inadequate selection and/or description of the non-exposed cohort and a small sample size.

### Intelligence

**Fig. 2:**
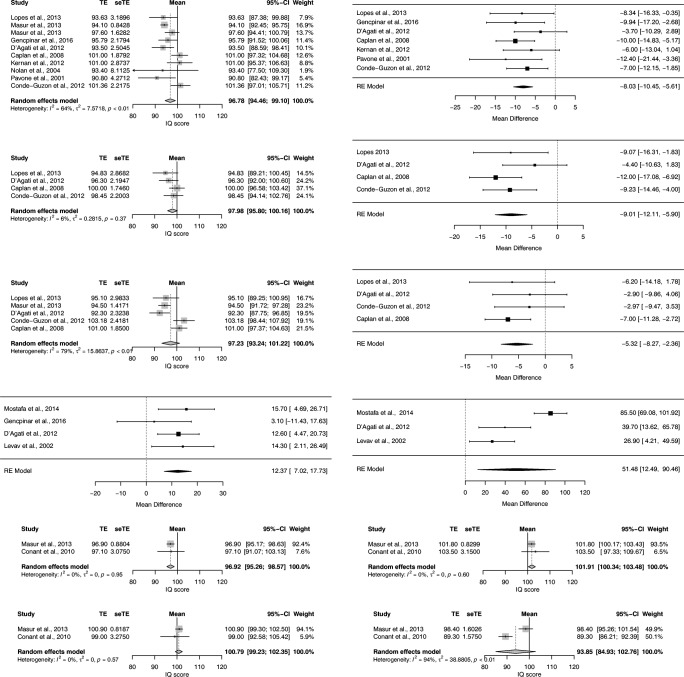
Forest plots of the single-arm meta-analyses (weighted average) and meta-analyses of the mean difference (difference in performance in case-control studies). For each study the mean is represented by a square (size is proportional to the study’s weight) and the 95% confidence interval (CI) is represented by a horizontal line. The overall weighted mean is represented by a diamond shape

Table 2 displays the neuropsychological results of the included study per cognitive domain. Wechsler Intelligence Test for Children (WISC-III) was the psychological test most often used to assess general intelligence. Of 11 studies reporting on full-scale IQ, nine studies were suitable for pooling of results (Caplan et al., 2008; Conde-Guzon & Cancho-Candela, 2012; D’Agati et al., 2012; Gencpinar et al., 2016; Kernan et al., 2012; Lopes et al., 2013; Masur et al., 2013; Nolan et al., 2004; Pavone et al., 2001). The estimated mean full-scale IQ from the single-arm random-effects meta-analysis was 96.78 (95% CI: 94.46–99.10; *T*^*2*^ = 7.57*:I*^*2*^ = 64.2%) for absence patients, which is significantly different from the normative mean of 100 (Fig. 2). The mean full-scale IQ based on a sensitivity analysis excluding studies with a moderate risk of bias was 97.43 (95% CI: 94.43; 100.43; *T*^*2*^ = 10.84; *I*^*2*^ = 75.9%). The estimated mean difference in IQ points compared to a control group in seven available case-control studies was −8.03 (95% CI: −10.45- -5.61; *T*^2^ = 0; *I*^*2*^ = 0%). The mean difference in IQ points in a sensitivity analysis excluding studies with a moderate risk of bias was −8.52 (95% CI:-11.31 - -5.73; *T*^*2*^ = 0; *I*^*2*^ = 0%). The estimated mean full-scale IQ of the control group was 105.09 (95% CI: 101.63–108.56; *T*^2^ = 17.40; *I*^*2*^ = 82%), which is significantly higher than the normative mean of 100.

**Table 2:** Neuropsychological test results of the included studies per cognitive (sub)domain

Cognitive (sub)domain	Reference	Test-version	Subtest/score	N in analysis	Results Absence Epilepsy Mean (SD)	Results Control group# Mean (SD)	P value or effect-size
Intelligence	Lopes et al., 2013	WISC-III	Full Scale IQ‡‡	30	93.63 (17.47)	101.97 (13.89)	NS
	Masur et al., 2013	WISC-IV		321 (≥6 years of age)	94.1 (15.1)		
		WPPSI–III		99 (<6 years of age)	97.6 (16.2)		
	Gencpinar et al., 2016	WISC-R		19	95.79 (9.50)	105.74 (13.05)	0.359
	D’Agati et al., 2012	WISC-III		15	93.5 (9.7)	97.2 (8.7)	0.177
	Caplan et al., 2008	WISC-R/WISC-III		69	101 (15.61)	111 (13.22)	< 0.0001*
	Kernan et al., 2012	WISC-III		31	101 (16)	107 (12)	NS
	Nolan et al., 2004	Stanford-Binet-IV/WISC-R/WISC-III		13	93.4 (29.25)		
	Pavone et al., 2001	WISC-R		13	90.8 (15.4)	103.2 (6.3)	< 0.01*
	Conde-Guzon & Cancho-Candela, 2012	WISC-R		34	101.36 (12.93)	108.36 (8.23)	NS
	Verrotti et al., 2011†	WISC-R/WPPSI-R		40	15% Decreased IQ		
	Franzoni et al., 2015†	WISC/WPPSI		92	27% Cognitive impaired		
	Lopes et al., 2013	WISC-III	Verbal IQ‡‡	30	94.83 (15.71)	103.90 (12.75)	NS
	D’Agati et al., 2012	WISC-III		15	96.3 (8.5)	100.7 (8.9)	0.217
	Caplan et al., 2008	WISC-R/WISC-III		69	100 (17.46)	112 (15.37)	< 0.0001*
	Conde-Guzon & Cancho-Candela, 2012	WISC-R		34	98.45 (12.83)	107.68 (7.9	0.021*
	Pavone et al., 2001†	WISC-R		13	94.4 (75–119)§	106.7 (84–117)§	NA
	Siren et al., 2007†	WISC-R/WPPSI-R		10	99.5 (84.0–128.0)§	94.0§	NS
	Nolan et al., 2004†	Stanford-Binet-IV/WISC-R/WISC-III		13	Normal to normative data		
	Lopes et al., 2013	WISC-III	Performance IQ‡‡	30	95.10 (16.34)	101.30 (15.19)	NS
	Masur et al., 2013	WPPSI-III		99 (≤6 years of age)	94.5 (14.1)		
	D’Agati et al., 2012	WISC-III		15	92.3 (9.0)	95.2 (10.4)	0.267
	Caplan et al., 2008	WISC-R/WISC-III		69	101 (15.35)	108 (11.85)	0.001*
	Conde-Guzon & Cancho-Candela, 2012	WISC-R		34	103.18 (14.1)	106.15 (11.99)	NS
	Pavone et al., 2001†	WISC-R		13	88.3 (65–115)§	101.9 (85–110)§	NA
	Siren et al., 2007†	WISC-R/WPPSI-R		10	96.0 (67–118)§	96.5 (74–102)§	NS
	Nolan et al., 2004†	Stanford-Binet-IV/WISC-R/WISC-III		13	Normal to normative data		
	Lopes et al., 2013	WISC-III	Verbal Comprehension Index‡‡	30	95.93 (15.50)	104.63 (12.52)	NS
	Masur et al., 2013	WISC-IV		321 (≥6 years of age)	93.1 (14.4)		
		WPPSI-III		99 (<6 years of age)	98.1 (16.4)		
	Schraegle et al., 2016	WISC-IV		30	89.5 (16.7)		
	Lopes et al., 2013	WISC-III	Processing Speed Index‡‡	30	95.27 (17.80)	102.57 (15.88)	NS
	Masur et al., 2013	WISC-IV		321 (≥6 years of age)	95.1 (15.6)		
		WPPSI-III		99 (<6 years of age)	96.3 (17.1)		
	Conant et al., 2010	WISC-III		16	104.4 (16.3)	115.7 (15.3)	−0.71¶
	Lopes 2013	WISC-III	Perceptual Organization Index‡‡	30	95.63 (17.20)	101.20 (14.64)	NS
	Masur 2013	WISC-IV		321 (≥6 years of age)	97.2 (15.1)		
	Masur 2013	WISC-IV	Working memory Index	321 (≥6 years of age)	94.8 (14.2)		
Non-verbal intelligence	Oostrom 2003	Coloured Progressive Matrices / Standard Progressive Matrices		10	99 (13)	101 (15)	NS
	Cheng 2017	Simplified Raven’s Progressive Matrices		43	13.21 (9.01)	17.69 (7.10)	< 0.05*
	Masur 2013	TONI-3		316 (≥6 years of age)	103.0 (14.2)		
Executive function	Masur 2013	WCST	Perseverative responses (standard score)	254 (≥6 years of age)	95.2 (15.0)		
	Gencpinar 2016	WCST	Perseverative responses	19	31.21 (16.59)	19.37 (9.35)	0.010*
			Perseverative errors		26.95 (12.23)	17.74 (8.66)	0.011*
			Nonperseverative errors		20.26 (9.99)	19.53 (9.35)	0.816
	Conant 2010	WCST	Perseverative errors (standard score)	16	80.1 (22.6)	98.0 (10.6)	−1.00 ¶
			Categories completed (standard score)		85.3 (17.3)	100.4 (14.3)B	−0.95 ¶
			Failure to maintain set (standard score)		106.9 (12.7)	98.1 (15.9)	0.61 ¶
	Kernan 2012	WCST	Total errors	31	38 (24)	30 (21)	< 0.05*
	Levav 2002	WCST	Perseverations responses	24	21.0 (10.6)	13.0 (10.2)	NA
			Perseverative errors		18.9 (8.8)	11.9 (9.1)	NA
			Categories completed		4.8 (1.5)	5.3 (1.2)	NA
			Failure to maintain set		1.2 (1.1)	0.6 (0.8)	NA
	Cheng 2017	WCST (adapted)		43	47.74 (28.01)	69.17 (15.62)	< 0.001*
	Gencpinar 2016	STROOP	Time	19	35.68 (12.78)	38.68 (12.92)	0.477
			Error		0.68 (1.49)	0.37 (0.76)	0.806
			Correction		1.42 (1.53)	1.53 (1.86)	0.879
	Kernan 2012	STROOP	Color Naming	31	65 (23)	53 (15)	NS
			Interference		50 (35)	43 (30)	NS
	Levav 2002	STROOP	Words	24	77.4 (19.6)	100.5 (20.6)	NA
			Colors		53.8 (14.6)	72.7 (13.9)	NA
			Color-Word		28.4 (9.7)	41.9 (12.8)	NA
	D’Agati 2012	Tower of London	Total score	15	27.6 (3.9)	29.7 (3.1)	0.160
			Total time		299.4 (97.2)	177.9 (64.3)	0.001*
	Conant 2010	Tower of London	Standard score	16	84.1 (17.2)	94.7 (13.8)	−0.68 ¶
	D’Agati 2012	COWAT	FAS - One min per letter- Total words	15	21.8 (4.3)	27.2 (4.1)	0.008*
	Henkin 2005	COWAT	FAS - Sum of all admissible words for F, A, S	12	10.2 (2.7)	12.6 (3.1)	NS
	Conant 2010	COWAT	One min per letter (standard score)	12	94.7 (19.3)	111.2 (15.9)	−0.94 ¶
	D’Agati 2012	CAT	Color, animals, fruits: Sum of total words	15	39.8 (8.8)	53.2 (7.7)	0.001*
	Henkin 2005	CAT	Animal-Food: Sum of two categories	12	16.7 (5.2)	20.6 (4.6)	< 0.05*
	Gencpinar 2016	CAT	Animal, Color, Fruits	19	18.68 (8.1)	26.00 (11.82)	.055
	Conant 2010	CAT	Animal: Standard score	16	98.5 (19.7)	108.3 (18.0)	−0.52 ¶
Attention	Masur 2013	CPT-II	Confidence Index	323	57.6 (25.5)		
			Omission T-score		<0.60 = 65%@ ≥0.60 = 11% >70 = 24%		
			Commission T-score		<0.60 = 93% ≥0.60 = 7% >70 = 0%		
		K-CPT	Confidence Index	85 (<6 years of age)	45.9 (18.6)		
			Omission T-score		<0.60 = 68% ≥0.60 = 20% >70 = 12%		
			Commission T-score		<0.60 = 78% ≥0.60 = 19% >70 = 4%		
	Levav 2002	CPT-X (Rosvold)	Correct	24	69.5 (10.0)	NA	NA
			Incorrect		7.0 (11.5)	NA	NA
			Reaction time		496.5 (96.9)	NA	NA
			Variability		99.6 (23.6)	NA	NA
		CPT-AX	Correct		61.2 (18.3)	73.5 (4.8)	NA
			Incorrect		15.7 (22.2)	1.4 (1.8)	NA
			A-non-X		2.7 (4.7)	0.4 (1.0)	NA
			Reaction Time		428.6 (100.8)	409.0 (68.4)	NA
			Variability		122.8 (24.9)	91.4 (31.1)	NA
		CPT-Deg. X	Correct		52.3 (18.5)	60.2 (11.9)	NA
			Incorrect		19.0 (11.9)	12.0 (14.2)	NA
			Reaction time		627.7 (102.1)	580.3 (69.3)	NA
			Variability		110.3 (23.8)	109.3 (27.3)	NA
		CPT-Tones	Correct		61.6 (15.9)	70.2 (10.7)	NA
			Incorrect		10.0 (9.8)	4.1 (7.4)	NA
			Reaction time		513.3 (100.9)	476.3 (72.1)	NA
			Variability		138.7 (19.8)	119.5 (57.8)	NA
		CPT-O	Correct		53.1 (18.1)	NA	NA
			Incorrect		10.7 (9.0)	NA	NA
			Reaction time		584.7 (105.1)	NA	NA
			Variability		138.3 (12.7)	NA	NA
		CPT-LO	Correct		61.8 (15.5)	64.5 (14.6)	NA
			Incorrect		9.3 (7.9)	4.6 (5.1)	NA
			Reaction time		486.0 (107.4)	475.8 (79.5)	NA
			Variability		136.2 (23.1)	121.5 (34.5)	NA
	Mostafa 2014	TMT A (Arabic)‡‡	Sustained attention	10	77.7 (16.2)	62 (12.6)	NA
		TMT B (Arabic)‡‡	Divided attention		199.6 (23.5)	114.1 (21.2)	NA
	Gencpinar 2016	TMT (Turkish)‡‡	Sustained attention	19	60.63 (25.96)	57.53 (19.25)	0.931
	D’Agati 2012	TMT A (adult)‡‡	Sustained attention	15	44.5 (13.5)	31.9 (8.7)	0.007*
		TMT B (adult)‡‡	Divided attention		146.1 (50.5)	106.4 (10.3)	0.041*
	Levav 2002	TMT A (age ≥ 13:adult)‡‡	Sustained attention	24	44.5 (29.5)	30.2 (11.6)	NA
		TMT B(age ≥ 13: adult)‡‡	Divided attention		99.5 (49.5)	72.6 (41.9)	NA
	Cerminara 2013	TAP, Tonic arousal	Reaction time (ms)	24	335.15 (101.63)	300.77 (45.83)	0.648
			Variability of reaction time (ms)		69.98 (58.93)	40.03 (17.63)	0.006*
			Number of omission errors		0.13 (0.45)	0.04 (0.20)	0.317
		TAP, Phasic arousal	Reaction time (ms)		309.17 (68.30)	287.08 (47.89)	0.271
			Variability of reaction time (ms)		90.12 (72.80)	50.23 (32.26)	0.009*
			Number of omission errors		0.13 (0.34)	0.29 (0.55)	0.157
		TAP, Vigilance	Reaction time (ms)	23	751.98(137.44)	809.15 (149.65)	0.338
			Variability of reaction time (ms)		174.64 (71.24)	178.24 (64.89)	0.951
			Number of commission errors		6.87 (7.09)	4.46 (3.72)	0.051
			Number of omission errors		6.13 (3.61)	5.21 (4.11)	0.603
		TAP, Divided attention	Reaction time (ms)	24	786.81 (113.50)	805.60 (86.73)	0.775
			Variability of reaction time (ms)		321.59 (95.49)	280.00 (87.73)	0.179
			Number of commission errors		4.21 (4.52)	2.50 (2.38)	0.264
			Number of omission errors		7.21 (3.73)	4.04 (3.37)	0.001*
		TAP, Impulsivity(Go/No-Go task)	Reaction time (ms)		666.67 (110.40)	634.44 (80.63)	0.126
			Variability of reaction time (ms)		132.94 (73.71)	91.45 (23.92)	0.006*
			Number of commission errors		2.67 (4.05)	0.63 (1.01)	0.001*
			Number of omission errors		1.00 (2.45)	0.04 (0.20)	0.028*
		TAP, Focused attention	Reaction time (ms)		533.56 (153.51)	556.17 (127.44)	0.290
			Variability of reaction time (ms)		193.89 (146.62)	156.62 (80.71)	0.607
			Number of commission errors		10.08 (9.19)	7.88 (7.17)	0.291
		TAP, Selective attention	Reaction time (ms)		3179.81 (1468.43)	4215.67 (1578.28)	0.004*
			Variability of reaction time (ms)		1938.79 (958.09)	2317.58 (1160.52)	0.199
			Number of commission errors		1.08 (2.38)	0.58 (1.10)	0.437
			Number of omission errors		6.17 (4.04)	3.96 (2.16)	0.020*
	Cheng 2017	Choice reaction time test	Reaction time in ms	43	532.74 (149.36)	435.28 (156.04)	< 0.01*
	Siren 2007	FePsy	Auditory reaction time Dominant hand (z-score)	6	- 1.15 (0.66)		NS
			Auditory reaction time Nondominant hand (z-score)		- 2.35 (1.47)		NS
			Visual reaction time Dominant hand (z-score)		- 2.19 (1.25)		NS
			Visual reaction time Nondominant hand (z-score)		- 0.29 (0.65)		NS
	Mostafa 2014	PASAT		10	19.1 (2)	19.5 (1.1)	NA
		Expressive Attention Task	Number of wrong answers		4.4 (1.8)	2.2 (1.3)	NA
		Receptive Attention Task (1)			31 (2.2)	31 (2.1)	NA
		Receptive Attention Task (2)	Number of wrong answers		3.4 (2)	1.9 (0.8)	NA
	Siren 2007	STIM tasks	Attention	8	81.5 (14.0–96.0)	80.5 (37.0–98.0)	NS
Language	Caplan 2008	TOAL or TOLD-2 primary/intermediate	Spoken language quotiënt	69	94 (17.16)	104 (13.31)	< 0.0001*
	Masur 2013	PPVT-III‡‡	Receptive vocabulary	310 (≥6 years of age)	99.3 (14.4)		
		PPVT-III‡‡		104 (<6 years of age)	99.5 (14.4)		
	Vanasse 2005	PPVT-R (French)‡‡		10	111.8	107.7	NS
	Vanasse 2005	Experimental Metaphonological awareness task (french)	Non-word repetition (% of correct responses)	10	83.8	83.75	NS
			Rhyme production (% of correct responses)		90.3	84.01	NS
			Phonemic blending (% of correct responses)		92.9	96.57	NS
			Phonemic segmentation (% of correct responses)		81.1	91.89	< 0.05*
			Phonemic inversion (% of correct responses)		83.1	92.87	NS
	Vanasse 2005	Denomination task (DEN 48)	Expressive language skills	10	78.1%	75.9%	NS
	Cheng 2017	Semantic comprehension	Sentence completion test	43	19.23 (11.33)	22.63 (14.62)	NS
		Word rhyming	Phonological processing ability		26.58 (8.80)	27.78 (9.98)	NS
	Henkin 2003	AERP	Tonal latency (N1, N2, P3)	12	‡	‡	NS
			Tonal amplitude (N1, N2, P3)		‡	‡	NS
			Phonetic latency “easy” (N1, N2, P3)		‡	‡	NS
			Phonetic amplitude “easy” (N1, N2, P3)		‡	‡	NS
			Phonetic latency “difficult” (N1, N2, P3)		‡	‡	NS
			Phonetic amplitude “difficult” (N1, N2, P3)		‡	‡	Only N2*
			Semantic latency (N1, N2, P3)		‡	‡	Only P3*
			Semantic amplitude (N1, N2, P3)		‡	‡	Only N2*
	Conde-Guzon 2012	LURIA-DNI	Phonemic hearing	34	‡	‡	< 0,001*
			Simple comprehension		‡	‡	NS
			Grammatical comprehension		‡	‡	NS
			Articulation & repitition		‡	‡	< 0,001*
			Denomination & narration		‡	‡	< 0,001*
			Phonetic		‡	‡	< 0,001*
Motor and							
Sensory-perceptual examinations	Conant 2010	Finger-tapping test	Dominant hand	16	103.5 (19.2)	108.7 (16.9)	−0.29 ¶
			Nondominant hand		102.2 (22.9)	112.1 (17.8)	−0.48 ¶
	Henkin 2005	Finger-tapping test	Right-hand finger tapping	12	36.2 (6.4)	42.9 (6.4)	< 0.05*
			Left-hand finger tapping		36.0 (6.9)	38.3 (6.4)	NS
			Right-left difference		0.16 (2.9)	4.6 (4.7)	< 0.05*
	Siren 2007	Finger-tapping test (STIM)	Dominant hand	10	39.8 (13.2–46.4)	42.3 (81.0–106.0)	NS
			Nondominant hand		34.3 (13.2–40.4)	35.5 (23.6–49.6)	NS
			Dominant/nondominant hand difference		3.6 (1.2–14.4)	5.9 (0.8–11.0)	NS
	Conant 2010	Complex Motor timing	Short interval 450 ms Variability-Syn.	13	44.1 (10.7)	41.9 (10.6)	−0.24 ¶
			Short interval 450 ms Variability-Con.		53.7 (16.3)	52.4 (16.2)	−0.09 ¶
			Long interval 750 ms Variability-Syn.		122.1 (34.3)	95.3 (32.8)	−0.70 ¶
			Long interval 750 ms Variability-Con.		121.0 (32.1)	88.0 (31.8)	−0.98 ¶
			Long interval 750 ms Mean ITI-Con.		666.1 (62.8)	719.2 (62.1)	−1.22 ¶
	Guerrini 2015	DGMP test	Prevalence of dysgraphia	82	21% (17/82)	8% (7/89)	0.016*
		Handwriting fluency test	Uno Test (Writing “Uno” for 1 min)		0.79 (95% CI-1.14,-0.45) (reduction of the z-score)		< 0.001*
			Le Test (Writing “Le” for 1 min)		1.32 (95% CI -1.72,-0.94) (reduction of the z-scores)		< 0.001*
	Conde-Guzon 2012	LURIA-DNI	Manual	34	‡	‡	< 0.001*
			Verbal regulation		‡	‡	NS
			Rhythm (Hearing sensory)		‡	‡	0,004*
			Tactile (Sensory)		‡	‡	< 0,001*
			Kinesthesia & stereognosis (Sensory)		‡	‡	NS
Visuoperceptual, visuospatial, and visuoconstructional function	Masur 2013	Beery-VMI‡‡	Visuomotor-integration	106 (<6 years of age)	98.4 (16.5)		
	Conant 2010	Beery-VMI‡‡		16	89.3 (6.3)	100.2 (11.3)	−1.20 ¶
	Conant 2010	KABC-HM	Visuomotor planning/integration	16	9.1 (3.4)	11.4 (2.8)	−0.74 ¶
	Henkin 2005	RCFT (Loring et al. 1988)	Copy	12	32.8 (3.2)	32.9 (3.8)	NS
	Nolan 2004	RCFT		13	Significantly worse to normative data		
	Pavone 2001	RCFT Fig. A and B		13	31.9 (percentile)	62 (percentile)	< 0.01*
	Cheng 2017	3D mental rotation test		43	14.44 (10.11)	19.39 (11.78)	NS
	Conde-Guzon 2012	LURIA-DNI	Spatial orientation	34	‡	‡	< 0,001*
			Visual perception		‡	‡	NS
	Levav 2002	Letter cancellation (raw test score)		24	51.6 (15.2)	70.8 (13.9)	NS
	Mostafa 2014	Visual search test (wrong)		10	4.3 (1)	1.9 (0.7)	NA
	Cheng 2017	Visual tracing (Grofman’s) (correct)		43	9.72 (6.40)	14.45 (7.19)	< 0.01*
Memory & Learning	D’Agati 2012	CBTT	Visuospatial short-term memory	15	4.6 (0.6)	5.0 (0.8)	0.338
	Lopes 2014	CBTT		30	8.97 (3.44)	9.85 (2.74)	NS
	Schraegle 2016	CVLT-C	Attention Span: A1	30	96.08 (14.32)		
			Attention Span: B1		94.85 (15.53)		
			Learning Efficiency: A5		98.85 (17.14)		
			Short Delay Free Recall: SDFR		99.05 (16.88)		
			Long Delay Free Recall: LDFR		98.63 (16.26)		
			Short Delay Cued Recall: SDCR		94.07 (15.92)		
			Long Delay Cued Recall: LDCR		94.85 (19.01)		
			Delayed Recognition		99.85 (13.01)		
			Inaccurate Recall: Total Intrusions on the delayed recognition task		97.15 (14.52)		
			Inaccurate Recall: False positives on the delayed recognition task		100.20 (15.61)		
	Kernan 2012	CVLT	Total (T-score)	31	51 (11)	56 (9)	< 0.05*
			Long-Delay Free (Z-score)		0.03 (1)	1 (1)	NS
			Discriminability (Z-score)		0.2 (1)	1 (1)	NS
	Henkin 2005	CVLT	Trial 1 (number of retrieved words)	12	6.6 (1.2)	8.2 (1.8)	< 0.05*
			Trial 3 (number of retrieved words)		10.7 (2.6)	13.1 (1.8)	≤ 0.01*
			Trial 5 (number of retrieved words)		12.6 (2.2)	14 (1.4)	NS
			Immediate recall (number of retrieved words)		10.7 (2.2)	13.1 (1.7)	≤ 0.01*
			Immediate cued recall (number of retrieved words)		10.7 (2.2)	14 (1.5)	≤ 0.001*
			Delayed recall (number of retrieved words)		11.2 (2.6)	13.9 (1.4)	≤ 0.01*
			Delayed cued recall (number of retrieved words)		11.2 (2.0)	14.2 (1.3)	≤ 0.001*
			Recognition (% correctly identified words)		96.5 (4.0)	99.1 (2.2)	NS
			Retrieval = subtraction of ‘delayed recall’ from ‘recognition’		4.2 (3.0)	1.9 (1.4)	< 0.05*
			Retention = substraction of ‘immediate recall’ from ‘delayed recall’		0.5 (1.9)	0.8 (1.4)	NS
	Gencpinar 2016	VADST – Form B	Aural-Oral	19	5.15 (0.95)	5.42 (1.34)	0.649
			Visual-Oral		5.21 (1.18)	5.31 (1.20)	0.880
			Aural-Written		4.94 (0.91)	5.57 (1.51)	0.105
			Visual-Written		5.15 (1.21)	5.42 (1.30)	0.641
	Lopes 2014	RCFT	Immediate recall	30	9.00 (3.27)	9.57 (2.67)	NS
			Delayed recall		8.63 (3.73)	9.59 (2.84)	NS
	Henkin 2005	RCFT (Loring et al. 1988)	Immediate recall	12	21.6 (5.1)	24.5 (7)	NS
			Delayed recall		21.1 (7.9)	23.2 (7.7)	NS
	Nolan 2004	RCFT	Recall	13	Significantly worse to normative data		
	Levav 2002	RAVLT	Total Learning over 5 trials	15	53.5 (8.2)	53.7 (8.6)	NA
			Learning rate T5-T1		5.0 (1.6)	5.8 (2.3)	NA
			Delayed memory (after 20 min)		12.2 (1.8)	11.1 (2.7)	NA
			Trial after interference list		11.5 (2.1)	11.4 (2.4)	NA
	Masur 2013	WRAML-2	Verbal Memory Index	319 (≥6 years of age)	99.1 (14.0)		
			Visual Memory Index		90.9 (15.6)		
	Conant 2010	WRAML	Screening Index (verbal + visual memory index)	16	106.0 (15.2)	105.0 (13.3)	0.07 ¶
			Picture Memory		12.1 (3.5)	9.7 (3.2)	0.71 ¶
			Design Memory		9.2 (2.9)	9.3 (3.1)	−0.03¶
			Verbal Learning		12.1 (3.2)	12.2 (2.4)	−0.04 ¶
			Story Memory		10.0 (2.7)	11.5 (2.9)	−0.54 ¶
			Verbal learning Delayed Recall		102.3 (15.6)	105.7 (13.1)	−0.24 ¶
			Story memory Delayed Recall		98.3 (13.6)	104.1 (15.3)	−0.40 ¶
			Story memory Recognition		104.8 (14.7)	106.9 (14.9)	−0.14 ¶
	Nolan 2004	WRAML	Verbal learning	13	Normal to normative data		
			Delayed verbal recall (verbal retention)		Normal to normative data		
			Story memory immediate		Normal to normative data		
			Delayed recall		Normal to normative data		
			Sentence memory		Normal to normative data		
			Visual Learning		Normal to normative data		
			Visual Retention		Normal to normative data		
			Picture memory		Normal to normative data		
			Design memory		Normal to normative data		
			Finger windows (visual memory)		Significantly worse to normative data		
	Gencpinar 2016	SDLT		19	13.31 (6.66)	17.31 (4.97)	0.037*
	Lopes 2014	LIST learning (words)	Learning	30	8.13 (3.28)	9.39 (2.82)	NS
			Immediate recall		8.70 (3.38)	9.38 (2.20)	NS
			Delayed recall		8.90 (3.07)	9.39 (2.27)	NS
			Recognition		8.20 (3.63)	10.25 (2.72)	NS
	Pavone 2001	TOMAL (Italian version)	Verbal Memory Index score (VMI)	13	76	80	NS
			Nonverbal Memory Index score (NMI)		49.9	71	< 0.05*
			Composite Memory Index (CMI)		65	69	NS
			Delayed Recall Index (DRI)		23.7	65	< 0.01*
	Kernan 2012	TOMAL	Memory for stories (standard score)	31	10 (3)	12 (3)	< 0.01*
			Memory for stories Delayed		9 (3)	11 (3)	< 0.01
	Siren 2007	STIM tasks	Visual memory	9	80.0 (46.7–94.7)	84.5 (47.4–100.0)	NS
			Spatial memory	10	96.7 (59.2–100.0)	93.8 (75.0–100.0)	NS
	Mostafa 2014	Memory tests	Spatial memory	10	7.2 (2.1)	8.2 (1.7)	NA
			Incidental (Verbal) memory test		8.1 (0.7)	8.4 (1.1)	NA
			Number Recall		7.7 (1.1)	9.8 (1.7)	NA
	Kernan 2012	Doors & People Verbal memory	Auditory Name Recall	31	20 (7)	23 (7)	NS
			Long-Delay Auditory Name Recall		8 (3)	9 (3)	NS
			Visual Name Recall		17 (5)	16 (6)	NS
			E–F Difference (Auditory Retention)		1 (2)	1 (2)	NS
		Doors & People Visual memory	Door Recall		18 (3)	17 (4)	NS
			Shapes Recall		31 (4)	32 (5)	NS
			Long Delayed Shapes		11 (1)	11 (2)	NS
			G–H Difference (Visual Retention)		0.4 (1)	0.2 (2)	NS
	Masur 2013	NEPSY-II	Sentence Repetition-Standard Score	104 (<6 years of age)	9.2 (3.0)		
	Conde-Guzon 2012	LURIA-DNI	Immediate (short-term) memory	34	‡	‡	< 0,001*
			Logical memory		‡	‡	< 0,001*
Achievement	Masur 2013	WRAT-3‡‡	Reading	318 (≥6 years of age)	101.8 (14.8)		
			Spelling		100.9 (14.6)		
			Arithmetic		96.9 (15.7)		
	Conant 2010	WRAT-3‡‡	Reading	16	103.5 (12.6)	107.7 (8.6)	−0.39 ¶
			Spelling		99.0 (13.1)	104.9 (6.1)	−0.57 ¶
			Arithmetic		97.1 (12.3)	102.3 (8.2)	−0.49 ¶
	Vanasse 2005	BELEC	Regular words	10	92.50% correct	99.17% correct	<0.05
			Irregular words		71.67% correct	84.17% correct	NS
			Regular words >1 y delay in school grade levels		40%	<10%	
			Irregular words >1 y delay in school grade levels		50%	<10%	
	Vanasse 2005	Non-word reading task	Non words	10	80.33% correct	87.75	NS
	Vanasse 2005	Alouette reading test	Mean reading age deficit in months	10	25.7 (14.28)	–	
	Cheng 2017	Simple substraction test		43	34.30 (12.02)	33.47 (9.94)	NS
	Conde-Guzon 2012	LURIA-DNI	Writing	34	‡	‡	0,009*
			Reading		‡	‡	0,001*
			Numerical structure		‡	‡	0,002*
			Arithmetic		‡	‡	0,029*
By proxy (parent/teacher reports)	Vega 2010	BASC subscale	Attention Problems (for subscores‡)	38	58.2 (12.7)	48.8 (9.3)	0.005*
	Shinnar 2017	CBCL	Attention	382	60.1 (9.78) 15% clinically significant>70		
	Caplan 2008	CBCL		69	37.5% clinically significant>60	15.50%	0.001*
	Conant 2010	CBCL		16	60.3 (8.1)	52.6 (5.0)	−1.14 ¶
	Shinnar 2017	Prevalence	ADHD	382	26%		
	Caplan 2008		ADHD	69	37%	8%	0.0002*
School difficulties	Urena-Hornos 2004	Prevalence	School problems	49	12 (24%)		
	Covanis 1992		Low average school performance	124	65/124 (52%)		
	Berg 2014		Special education services prior to diagnosis	57	13/57 (23%)		
	Oostrom 2003		Special educational assistence	10	7/10 (70%)		
Neuropsychological or neurodevelopmental problems	Berg 2011	Prevalence	Ever presence of: developmental delay, learning disorder, mental retardation, autism spectrum disorder, auditory processing disorder, dyslexia	51	13/51 (25,5%)		
	Fastenau 2009		Neuropsychological deficit in at least one domain ‡	38	31,6%		
	Sinclair 2007		Intellectual disability	119	26/119 (22%) Typical AE 13/80 (16%) Atypical AE 13/39 (33%)		
	Wirrell 1996		Cognitive difficulties at presentation (reported by parent/teacher/physician)	58	14/58 (24%)		

AERP, Auditory Event-Related Potential Test; ADHD, Attention Deficiency Hyperactive Disorder; BASC, Behavior Assessment System for Children; BELEC, Belgian Reading Battery; CAT, Category Fluency Test; CBCL, Child Behavior Checklist; CBTT, Corsi Block Tapping Test; COWAT, Controlled Oral Word Association Test; CPT, Continuous Performance Test; CVLT, California Verbal Learning Test; DGMP, Graph-motor and posture disorders of handwriting test; KABC, Kaufmann Battery for Children; N, number of patients; NA, Not Available; NS, Not Significant; PASAT, Paced Auditory Serial Addition Test; PPVT, Peabody Picture Vocabulary Test; RAVLT, Rey Auditory Verbal Learning Test; RCFT, Rey-Osterrieth Complex Figure Test; SD, Standard Deviation; SDLT, Serial Digit Learning Test; TAP, Computerized Test Of Attentional Performance; TMT, Trail Making Test; TOAL, Test of Adolescent Language; TOMAL, Test of Memory and Learning; TOLD, Test of Language Development; TONI-3, Test of Nonverbal Intelligence-3; VADST, Visual Aural Digit Span Test; WCST, Wisconsin Card Sorting Test; WISC, Wechsler Intelligence Scale for Children; WRAML, Wide Range Assessment of Memory and Learning; WPPSI, Wechsler Preschool and Primary Scale of Intelligence; WRAT, Wide Range Achievement Test

* Significant, † Not included in meta-analysis, ‡ Refer to original paper, § Median, ¶ Cohen’s d, ‡‡ Meta-analysis available

Out of seven studies reporting on verbal IQ, four could be included in the meta-analysis with an estimated mean of 97.98 (95% CI: 95.80–100.16; *T*^2^ = 0.28; *I*^2^ = 5.5%) and an estimated mean difference of −9.01 (95% CI: −12.11- -5.90; *T*^2^ = 1.31; I^2^ = 12.85%) compared to controls (Fig. 2).

Out of seven studies reporting on performance IQ, four were suitable for meta-analytic comparison with an estimated mean of 97.23 (95% CI: 93.24–101.22; *T*^2^ = 15.86; *I*^2^ = 78.7%) and an estimated mean difference of −5.32 (95% CI: −8.27-2.36; *T*^2^ = 0; I^2^ = 0%) compared to controls (Fig. 2).

The estimated mean for the Verbal Comprehension Index was 94.43 (95% CI: 91.11–97.75; *T*^2^ = 7.39; *I*^2^ = 70.2%), the estimated mean for the Processing Speed Index was 96.30 (95% CI: 93.74–98.86; *T*^2^ = 2.77; *I*^2^ = 41.9%) and the estimated mean for the Perceptual Organization Index was 97.09 (95% CI: 95.50–98.69; *T*^2^ = 0; *I*^2^ = 0%). One study, which used the WISC-IV, reported a mean Working Memory Index of 94.8 (SD 14.2). Sensitivity analyses by excluding outliers based on the funnel plots did not yield different conclusions. Results on the subtests of the Wechsler Intelligence tests are available in Table S1. Non-verbal intelligence tests were used in three studies. In the study with by far the largest sample of Masur et al. (*n* = 316) non-verbal intelligence assessed with the TONI-3 test was significantly higher compared to the normative mean (Masur et al., 2013). Raven’s Progressive Matrices was used by Oostrom et al. which reported no significant difference from normative values in a small sample (*n* = 10) of children with either CAE or JAE (2003). A simplified version of the Raven’s Progressive Matrices was used by Cheng et al. in children with CAE (*n* = 43) which performed significantly worse compared to controls (2017).

### Executive Function

A total of eight studies assessed executive function (Cheng et al., 2017; Conant et al., 2010; D’Agati et al., 2012; Gencpinar et al., 2016; Henkin et al., 2005; Kernan et al., 2012; Levav et al., 2002; Masur et al., 2013) using different instruments. The performance of children with AE on the STROOP test did not significantly differ from controls (Gencpinar et al., 2016; Kernan et al., 2012; Levav et al., 2002). Significant worse performance was noted in multiple studies using the Wisconsin Card Sorting Test (Cheng et al., 2017; Conant et al., 2010; Gencpinar et al., 2016; Kernan et al., 2012; Levav et al., 2002; Masur et al., 2013), the Category Fluency Test (Conant et al., 2010; D’Agati et al., 2012; Henkin et al., 2005) and the Tower of London Test (Conant et al., 2010; D’Agati et al., 2012). In two out of three studies lower function was noted on verbal fluency with the Controlled Oral Word Association Test (Conant et al., 2010; D’Agati et al., 2012; Henkin et al., 2005). The control group in the study of Conant et al. scored higher than the normative mean; while the children with AE scored approximately average. Pooling of results was not possible for these tests due to differences in administration of the neuropsychological test and/or outcome reporting.

### Attention

A total of eight studies assessed attention (Cerminara et al., 2013; Cheng et al., 2017; D’Agati et al., 2012; Gencpinar et al., 2016; Levav et al., 2002; Masur et al., 2013; Mostafa et al., 2014; Siren et al., 2007). A large randomized clinical trial in CAE showed impairments in attention in up to 1/3 of patients with the Conner’s Kiddie Continuous Performance Test in patients just started with anti-epileptic drug monotherapy (<1 week) or before starting treatment (Masur et al., 2013). Errors of omission (missing relevant targets) were more common than errors of commission (responding to non-targets). During follow-up, attentional deficits persisted independent of anti-epileptic drug treatment or seizure control. The study of Levav et al. used the Rosvold Continuous Performance test and also reported diminished scores in visual sustained attention (2002). However, in this study, the mean age of the control group used was 12 years older than the group with AE.

On the Trail Making Test A, the pooled estimated mean difference compared to controls was 12.37 s (95% CI: 7.02–17.73; *T*^2^ = 0; *I*^2^ = 0%) longer for children with AE and on the Trail Making Test B an estimated mean difference was found of 51.48 s (95% CI: 12.49–90.46; *T*^2^ = 1061.59; *I*^2^ = 89.9%) (Fig. 2) (Conant et al., 2010; D’Agati et al., 2012; Levav et al., 2002; Mostafa et al., 2014).

Furthermore, Cerminara et al. found significantly lower scores in some measures of alertness, divided attention, impulsivity, and selective attention in CAE compared to controls (2013). The divided and selective attention tasks were characterized by more errors of omission, whereas the impulsivity task was characterized by more commission errors. Reaction times had significantly more variability during the tonic arousal, phasic arousal and impulsivity task, but not in the divided or selective attention task. Focused attention did not differ from controls; however, Cheng et al. (2017) did find a significantly longer choice reaction time, which is similar to the focused attention task in the study performed by Cerminara et al. (2013).

Mostafa et al. reported significantly lower mean scores compared to controls in an expressive attention and receptive attention task; however, it was not clear whether this was still statistically significant when corrected for the 6 years age difference with controls (2014). Siren et al. used FEPSY auditory/visual reaction times and STIM tasks to assess attention and did not find a significant difference in this small sample (*n* = 10) (2007).

### Language

A total of six studies assessed language (Caplan et al., 2008; Cheng et al., 2017; Conde-Guzon & Cancho-Candela, 2012; Henkin et al., 2003; Masur et al., 2013; Vanasse et al., 2005). Caplan et al. reported significantly lower scores on the Spoken Language Quotiënt (SLQ) using the Test of Language Development (TOLD) compared to a control group (2008). Vanasse et al. used a metaphonological awareness task and reported significant worse phonemic segmentation compared to controls (2005). None of the other variables in this test differed from controls.

Masur et al. and Vanasse et al. did not find a significant difference in receptive vocabulary using the Peabody Picture Vocabulary Test (PPVT) compared to normative values (Masur et al., 2013; Vanasse et al., 2005). Vanasse et al. additionally used a denomination test to assess expressive language in a small sample of 10 subjects, but this did not yield a significant difference (2005).

The study of Conde-Guzon and Cancho-Candela in typical AE patients reported significantly lower performance in phonemic hearing, articulation/repetition, denominating/narration and phonetic analysis (2012). Comprehension and understanding of simple grammar did not differ significantly.

In a recent study by Cheng et al. semantic comprehension and word rhyming was not significantly worse compared to controls (2017). Furthermore, Henkin et al. reported on auditory event-related potentials and found significant increased N2 amplitudes for phonetic and semantic processing, as well as, a significantly increased latency for semantic stimuli for P3 compared to controls (2003).

### Motor and Sensory-Perceptual Examinations

Five studies have investigated motor function (Conant et al., 2010; Conde-Guzon & Cancho-Candela, 2012; Guerrini et al., 2015; Henkin et al., 2005; Siren et al., 2007). Siren et al. performed a finger-tapping test in the dominant and non-dominant hand but did not find significant differences compared to controls (2007). Conant et al. also reported normal fine motor speed using a finger tapping test in the dominant and non-dominant hand (2010). The control group in the study of Conant et al. scored higher than the normative mean; while children with AE scored approximately average. Conant et al. also assessed complex motor control and reported worse performance in AE compared to controls. Pooling of results with the finger tapping test was not possible due to differences in test protocols and reporting of results. Contrarily, Henkin et al. did find a significantly lower amount of taps per trial with finger-tapping in the right hand but not in the left-hand (2005).

Furthermore, Guerrini et al. reported a higher prevalence of dysgraphia in AE (21% vs. 8% for controls) and reported diminished overall performance in a handwriting fluency test compared to controls (2015). Conde-Guzon and Cancho-Candela used the LURIA-battery to assess motor and sensory functions compared to control subjects (2012). They reported significantly lower performance in the manual subtest, but verbal regulation did not differ from normative values. Sensory functions such as rhythmic hearing and tactile subtests were significantly worse compared to controls, while kinesthesia and stereognosis did not differ from normative values.

### Visuoperceptual, Visuospatial, and Visuoconstructional Function

Two studies assessed visuomotor planning and integration. Of the studies using the Beery-VMI, the largest study from Masur et al. (*n* = 106) reported a mean of 98.4 (SD 16.5), which is well within normal limits in children with CAE younger than 6 years of age (2013). Conant et al. included a smaller subset of patients (*n* = 16) and reported a significantly lower score of 89.3 (SD 6.3) compared to controls (2010). The single-arm random effect meta-analyses for the Beery-VMI was estimated at a mean of 93.85 (95% CI: 84.93–102.76; *T*^2^ = 38.88; *I*^2^ = 93.9%), which is not significantly different from a normative mean of 100 (Fig. 2).

On the contrary, Conant et al. found significant lower performance using the KABC-HM (imitation of hand movements) a test for visuomotor planning and integration (Conant et al., 2010).

Five studies have investigated visuospatial skills. In total three studies used the Rey-Complex Test (RCFT). Nolan et al. found significant worse performance in the RCFT compared to normative data but did not include the average scores (2004). Pavone et al. found lower performance in the RCFT compared to the control group, although still within normal clinical range (2001). Henkin, et al. did not find a significant difference in the RCFT with the control group (2005). Cheng et al. (2017) reported that children with CAE did not perform worse in a 3D mental rotation test, however, Conde-Guzon and Cancho-Candela did find significant worse performance in the visuospatial subtest of the LURIA-DNI neuropsychological battery (2012).

Two studies assessed visual search ability. Levav et al. used a Letter Cancellation test and reported a large difference in completing the test compared to controls, however, the age difference between patients with CAE and controls was ~12 years (2002). In a study by Mostafa et al. total duration of a Visual Search Test was significantly worse compared to controls, however, controls were 6 years older on average (2014).

### Learning and Memory

Fourteen studies assessed memory function (Conant et al., 2010; Conde-Guzon & Cancho-Candela, 2012; D’Agati et al., 2012; Gencpinar et al., 2016; Henkin et al., 2005; Kernan et al., 2012; Levav et al., 2002; Lopes et al., 2014; Masur et al., 2013; Mostafa et al., 2014; Nolan et al., 2004; Pavone et al., 2001; Schraegle et al., 2016; Siren et al., 2007). Pavone et al. used the Test Of Memory And Learning (TOMAL) and found significant impairments in the Nonverbal Memory Index Score, Delayed Recall Index and Kernan et al. found significant differences in the Memory for Stories Subtest Scores compared to control subjects (Kernan et al., 2012; Pavone et al., 2001).

Schraegle et al. reported intact verbal memory and list learning compared to normative data using the California Verbal Learning Test (auditory memory) (2016). However, Kernan et al. (2012) and Henkin et al. (2005) did find significant memory impairment using this test compared to control subjects. Kernan et al. reported a significant difference in the total mean score, whereas Henkin et al. reported significant differences in immediate recall, delayed recall, and retrieval.

Findings on the recall of the Rey-Osterrieth Complex Figure Test (visual memory) did not differ compared to controls, although Nolan et al. reported significant worse performance compared to normative data (Henkin et al., 2005; Lopes et al., 2014; Nolan et al., 2004).

In the Randomized Clinical Trial of Masur et al. the Wide Range Assessment of Memory and Learning (WRAML) test showed a normal Verbal Memory Index, whereas the Visual Memory Index was abnormal (2013). In addition, Conant et al. observed the largest differences between children with AE and controls in picture memory and story memory subtests of the WRAML test (2010). Nolan et al. reported normal scores compared to normative data for all subtest of the WRAML test in a small study (*n* = 13) (2004).The Serial Digit Learning Test used by Gencpinar et al. revealed a significant difference compared to controls (Gencpinar et al., 2016). Number recall also seems diminished in the study of Mostafa et al., however controls were on average 6 years older (Mostafa et al., 2014).

Lopes et al. reported normal performance on LIST learning for words (2014). Masur et al. reported a Sentence Repetition mean standard score of 9.2 which is significantly lower compared to a normative mean of 10 in children with CAE younger than 6 years of age (2013). Conde-Guzon and Cancho-Candela reported impairments in immediate (short-term) memory and logical memory in typical absence epilepsy compared to controls (2012). The performance on several other memory tests (Corsi Block Tapping Test (D’Agati et al., 2012; Lopes et al., 2014), Visual Aural Digit Span Test (Gencpinar et al., 2016), Rey Auditory Verbal Learning Test (Levav et al., 2002), Spatial Memory Test (Mostafa et al., 2014), Incidental Verbal Memory Test (Mostafa et al., 2014), STIM-tasks (Siren et al., 2007) and Doors and People (Kernan et al., 2012) did not differ in performance from control subjects.

### Achievement

Five studies used an achievement test (Cheng et al., 2017; Conant et al., 2010; Conde-Guzon & Cancho-Candela, 2012; Masur et al., 2013; Vanasse et al., 2005). Masur et al. (2013) and Conant et al. (2010) reported on arithmetic, reading and spelling ability using the Wide Range Achievement Test-3. On arithmetic, the estimated mean was 96.92 (95% CI: 95.26–98.57; *T*^2^ = 0; *I*^2^ = 0%) (Fig. 2). On spelling, the estimated mean did not differ from normative data 100.79 (95% CI: 99.23–102.35; *T*^2^ = 0; *I*^2^ = 0%). However, the estimated mean on reading was 101.91 (95% CI: 100.34–103.48; *T*^2^ = 0; *I*^2^ = 0%). Vanasse et al. investigated reading ability in a small group (*n* = 10) and found lower scores across a regular, irregular and non-word reading task, albeit only the regular word reading task was significantly different from the control group (2005). The post-hoc administered Alouette reading task showed a mean reading deficit of 25.7 months (SD 14.28) in children with CAE.

In children with CAE, 40–50% had more than 1-year delay in school grade levels compared to 10% of the control group. Conde-Guzon and Cancho-Candela found significant worse performance in writing, reading, numerical structure and arithmetic abilities compared to controls using the LURIA-DNI battery (2012).

### Reports on Attention or Attentional Deficiencies (by Proxy)

Vega et al. reported attentional problems, especially forgetfulness and distractibility to be more prevalent in children with CAE compared to controls using the Behavior Assessment System for Children (2010).

The prevalence of attentional problems in 38% of children with AE differed significantly compared to 16% in control subjects assessed with the Child Behavior Checklist (CBCL) in the study by Caplan et al. (2008). However, Shinnar et al. (2017) reported a lower percentage of 15% clinically significant attentional problems in CAE compared to the study of Caplan et al. (2008) but used a higher cut-off value. Conant et al. (2010) and Shinnar et al. reported similar mean scores on attention, which in the study of Conant et al. was significantly higher than controls. Caplan et al. also reported a higher prevalence of 37% with a diagnosis of attentional deficit hyperactivity disorder (ADHD) versus 6% in control subjects in their population (2008). Shinnar et al. estimated a 26% prevalence of ADHD in drug-naïve children with CAE (2017).

### Prevalence of School Difficulties

Four studies reported on school performance (Berg et al., 2014; Covanis et al., 1992; Oostrom et al., 2003; Urena-Hornos et al., 2004). Urena-Hornos et al. reported school problems in 12 out of 49 (24%) children with AE using a telephonic follow-up assessment (2004). Covanis et al. reported a low average school achievement in 65 out of 124 (52%) children with CAE (1992). Berg et al. reported that 13 out of 57 (23%) CAE patients had already received special education prior to diagnosis of epilepsy (2014). Oostrom et al. reported special educational assistance in 7 out of 10 children with either CAE or JAE (2003).

### Miscellaneous Data on Neurodevelopmental Problems

Four studies assessed the prevalence of neuropsychological and/or neurodevelopmental problems (Berg et al., 2011; Fastenau et al., 2009; Sinclair & Unwala, 2007; Wirrell et al., 1996). In the study of Berg et al., neurodevelopmental disorders (Table 2) were present in 13 out of 51 (26%) patients (2014). Fastenau et al. reported that 32% of 38 patients had at least one neuropsychological deficit in at least one domain (see Table 2) (2009). Sinclair & Unwala reported intellectual disability in 22% out of 119 children with CAE. Subgroup analysis showed intellectual disability in 16% of children with typical absences and 33% of children with AE and additional atypical features (2007). Wirrell et al. reported cognitive difficulties at presentation in 24% out of 58 children with AE (1996).

## Discussion

The aim of this review was to systematically assess the literature on cognitive performance in AE. Children with AE are regarded to have cognitive functioning within normal range (Adie, 1924; Currier et al., 1963). Nevertheless, we found multiple studies reporting lower cognitive performance across a wide spread of cognitive domains. However, the exact degree of impaired cognitive functioning is difficult to estimate as the methodologies across studies vary and multiple neuropsychological tests have been used, which hampers comparisons between studies. Moreover, it is currently difficult to distinguish momentary effects on cognitive performance during the active stage of AE from long-lasting effects on cognitive functioning, as most studies reported on cognitive performance at different time-points after seizure onset. Some studies tested prior to introduction of anti-epileptic drug treatment, while others reported on a mixed population of children on or off anti-epileptic drug treatment and with or without ongoing seizures.

### Intelligence

Full-scale IQ was estimated to be approximately three points lower on average compared to normative values. It is important to note that, although average performance in intelligence measures are statistically lower, the pooled averages still fall well within normal values. However, in case-control studies, the mean difference is larger with a difference of ~8 points in full-scale IQ, ~9 points in verbal IQ and ~5 points in performance IQ. There may be several explanations for these results. The mean IQ of the control subjects was significantly higher than normative values, which may simply resemble a higher average IQ in the studied population or geographical area. However, it may be due to exclusion of patients with a low IQ in several case-control studies (Caplan et al., 2008; D’Agati et al., 2012; Gencpinar et al., 2016; Henkin et al., 2005; Kernan et al., 2012; Lopes et al., 2013). Another explanation for a higher IQ in controls may be bias due to convenience sampling (e.g. children from academics), although this was not evident based on the methods used for the recruitment of control subjects in these studies. However, the estimated mean IQ in the single-arm meta-analysis in patients with AE is less subject to bias due to sampling error as a far larger proportion of the total population is being tested. Moreover, the estimated true variance (*T*^*2*^*)* for the single-arm meta-analyses was totally dependent on the three studies (Caplan et al., 2008; Conde-Guzon & Cancho-Candela, 2012; Kernan et al., 2012) with the highest mean IQ scores in AE and controls. The estimated true variance (*T*^*2*^*)* was small for the pooled difference in case-control studies.

### Executive Function

Lower than average performance in executive functioning was noted in cognitive flexibility, planning and verbal fluency (Cheng et al., 2017; Conant et al., 2010; D’Agati et al., 2012; Gencpinar et al., 2016; Henkin et al., 2005; Kernan et al., 2012; Levav et al., 2002; Masur et al., 2013). However, not all tests were indicative of lower executive functioning as results on the STROOP test did not differ.

### Attention

There are clear indications for a lower performance in attention, such as sustained attention, selective attention and divided attention. From our meta-analysis, we can conclude that trail making A and B scores take significantly more time to perform by children with AE compared to controls, especially when attentional shifts (divided attention) are necessary. The estimated true variance (*T*^*2*^*)* for the trail making test B was high, however, this may be explained by differences in study design, as the study by Levav et al. reported in children ≥13 years of age, which would probably require less seconds to finish the test than younger counterparts in the other two studies. Furthermore, in the study by Masur et al. sustained attention was mostly affected due to attentional lapses (errors of omission) rather than reflecting disinhibition (errors of commission) (2013).

### Language

Results on specific language tests are of particular interest, as verbal IQ was estimated to be lower in case-control studies, and a relatively low mean verbal comprehension index (in children >6 years of age) was reported in the study by Masur et al. (2013). However, receptive vocabulary was not affected with the Peabody Picture Vocabulary Test in the study by Masur et al. (Masur et al., 2013). Two studies with a decent sample size raise concerns regarding expressive language as found by Caplan et al. (although average functioning is still within normal clinical range) and Conde-Guzon & Cancho-Candela (Caplan et al., 2008; Conde-Guzon & Cancho-Candela, 2012). However, these two studies were characterized by relatively high verbal IQ’s in controls. Therefore, data on language tests in AE remains inconclusive, but warrants further research.

### Motor Function

Studies on simple motor tasks were inconclusive (Conant et al., 2010; Henkin et al., 2005; Siren et al., 2007). Complex motor tasks may be impaired but were only assessed in one study (Conant et al., 2010) and the control group was characterized by higher scores than the normative mean. Furthermore, one study found a higher prevalence of dysgraphia and diminished performance in handwriting fluency. These findings were associated with abnormal neurophysiological findings, which led the authors to conclude that these patients had a form of dystonic dysgraphia. Interestingly, 12 out of 17 patients with initial dysgraphia were re-tested 5-years later and showed resolution of dysgraphia and improved handwriting skills. In addition, Conde-Guzon and Cancho-Candela also reported worse performance in a writing test (2012).

### Visuoperceptual, Visuospatial, and Visuoconstructional Function

Visual-motor integration from the Beery-VMI test did not differ from the normative mean in our meta-analysis, although only two studies were available. The lower mean score in the Beery-VMI in the study by Conant et al. may be due to a small study effect. Studies on visual search tests and visuospatial skills remain inconclusive (Cheng et al., 2017; Conde-Guzon & Cancho-Candela, 2012; Henkin et al., 2005; Levav et al., 2002; Mostafa et al., 2014; Nolan et al., 2004; Pavone et al., 2001). Indeed, visual-spatial thinking ability in the meta-analysis of Loughman et al. in idiopathic generalized epilepsies did also not differ significantly in case-control studies (2014).

### Learning and Memory

Results on memory have yielded inconclusive results. The largest study on memory by Masur et al. suggests lower visual memory function, although still within normal clinical range (2013). However, other studies on non-verbal memory tests have overall found average performance (Conant et al., 2010; D’Agati et al., 2012; Gencpinar et al., 2016; Henkin et al., 2005; Kernan et al., 2012; Lopes et al., 2014; Nolan et al., 2004; Pavone et al., 2001; Siren et al., 2007). Furthermore, studies on verbal memory mostly showed normal performance (Levav et al., 2002; Lopes et al., 2014; Schraegle et al., 2016). Nevertheless, some studies using subtests for memory for stories and with the California Verbal Learning Test did find lower performance compared to controls (Conant et al., 2010; Henkin et al., 2005; Kernan et al., 2012). However, the observed differences with controls in these studies might suggest a sample bias related to a higher functional level of the controls. Only, one study found an overall lower than average performance in memory functioning, especially in short-term auditory, visual memory and logical memory, although this study was also potentially characterized by a better than average control group as reflected by the performance of the control group on the WISC-R (Conde-Guzon & Cancho-Candela, 2012). Sentence repetition was lower compared to the normative mean in the study by Masur et al. (2013), although scores still fall within normal clinical range and may be related to attentional deficits.

### Achievement

Arithmetic ability may be vulnerable based on our meta-analysis of the WRAT-3 test, although test scores fall within the normal clinical range. Nevertheless, arithmetic ability was also worse compared to controls in children with AE in the study by Conde-Guzon and Cancho-Candela (2012). Evidence regarding reading ability, is contradictory, as the meta-analysis on the WRAT-3 test was not significantly lower, but other reading tests by Vanasse et al. (2005) and Conde-Guzon & Cancho-Candela (2012) did report worse performance compared to controls.

### By Proxy (Parent-Reported Functioning)

Studies using parental questionnaires also point towards attentional problems and a higher prevalence of attentional deficit hyperactivity disorder.

### Prevalence of School Difficulties

School difficulties seem more prevalent in patients with AE than in the normal population, as the three largest studies reported school difficulties in 23% to 52% (Berg et al., 2014; Covanis et al., 1992; Urena-Hornos et al., 2004).

### Prevalence of Neuropsychological and/or Neurodevelopmental Problems

A prevalence of neuropsychological and/or neurodevelopmental problems were found in approximately 22% to 32% of the patients with CAE (Berg et al., 2014; Fastenau et al., 2009; Sinclair & Unwala, 2007; Wirrell et al., 1996).

### General Discussion

Cognitive deficits in one area may be related to the performance in another cognitive domain. Masur et al. reported a direct sequential effect among attention, memory, executive function, and academic achievement (2013). Therefore, the emergent pattern of clear attentional deficits in a proportion of children with absence epilepsy may influence performance in other cognitive domains. Nevertheless, the overall pattern suggests vulnerabilities in intelligence, attention and executive function. Less conclusive results were found for (expressive) language, motor function, visuo-perceptual functioning and learning & memory. Ultimately, vulnerabilities in cognitive domains may impact neurocognitive development and lead to more academic difficulties.

How children with AE mature into adulthood is far less researched. The few studies that have investigated cognitive function during adulthood in patients with AE suggest that lower performance on neuropsychological tests may persist, but current studies are small in sample size. A study in (*n* = 10) adults with CAE in remission reported a full-scale IQ of 92 (69–99), a performance IQ of 85 (66–117), with particularly low scores in the picture arrangement, block design and object assembly subtests of the WAIS-R (Hommet et al., 2001). Language and executive function were also tested using specific tests, but did not differ with a control group. Recently Loughman et al. reported on cognitive function using the Woodcock-Johnson-III test of cognitive abilities in an adult population of genetic generalized epilepsy syndromes, including CAE (n = 10) and JAE (*n* = 21) patients (2017). This study reported lower scores on brief intellectual ability, crystallized intelligence, new learning/memory and speed of processing (Loughman, Bowden, & D'Souza, 2017). In conclusion, neurodevelopment may ultimately differ in AE, as a study also found abnormal cortical thickness connectivity in various regions after in young adults with a history of CAE (Curwood et al., 2015).

What the impact may be on their working careers and psychosocial well-being is another important question. In a telephone follow-up study, 25% of 52 retrospectively identified patients of 11 to 36 years old with CAE had a history of psycho-pedagogical help (Martinez-Ferrandez et al., 2017). Another study in patients focusing on long-term psychosocial outcome in juvenile myoclonic epilepsy (JME) with a mean age over 60 years used AE patients as an epilepsy matched-control group (Holtkamp, Senf, Kirschbaum, & Janz, 2014). To the surprise of the authors, a significantly lower amount of patients with a history of AE accomplished a university degree compared to controls. Nevertheless, the overall psychosocial outcome of this study was favorable. However, another study compared a group of patients with rheumatoid arthritis to patients with CAE and similar impact of “chronic disease” (Camfield & Camfield, 2007). Young adults with a history of CAE were more prone to working in jobs requiring minimum education, having more behavioral problems and psychiatric consultations. Therefore, the potential impact later on in life cannot be underestimated and deserves further scientific attention.

Cognitive findings may also be related to (inter)ictal activity, pharmacological effects or another underlying (genetic) vulnerability, but disentangling all factors may not be feasible. The use of valproate was associated with attentional dysfunction in the study by Masur et al. (Cnaan et al., 2017; Dlugos et al., 2013; Glauser et al., 2010; Masur et al., 2013). In addition, seizure duration of more than 20 s correlated well with attentional dysfunction and errors of omission (Dlugos et al., 2013). In the prospective cohort study of Caplan et al. verbal IQ was associated with duration of illness and AED treatment. Moreover, ADHD was associated with the duration of illness and seizure frequency (2008). However, these associations have not been consistent across the literature.

Insights into possible underlying mechanisms are starting to be unraveled in recent years. Multiple studies using advanced magnetic resonance imaging (MRI) techniques in AE have for example found differences in brain regions or networks which are associated with executive function (Bai et al., 2011; Berman et al., 2010; Caplan et al., 2009; Carney, Masterton, Flanagan, Berkovic, & Jackson, 2012; Curwood et al., 2015; Li et al., 2015; Luo et al., 2014; Tosun, Siddarth, Toga, Hermann, & Caplan, 2011; Zhang et al., 2014), attention (Berman et al., 2010; Killory et al., 2011; Li et al., 2015; Luo et al., 2011; Luo et al., 2014; Zhang et al., 2014) and expressive language (Bai et al., 2011; Caplan et al., 2009; Curwood et al., 2015; Holmes, Brown, & Tucker, 2004; Luo et al., 2011; Tosun et al., 2011; Tucker, Brown, Luu, & Holmes, 2007).

### Limitations

We opted to use single-arm meta-analyses and/or a meta-analysis of the difference between cases and controls. This was chosen as sampling error may be of greater influence in a relatively small sample of the normal population, than it would be to estimate the true mean in AE, as a larger proportion of the group is likely to be included in a given geographical area. In addition, some studies did not match a control group specifically to children with AE as other types of epilepsy were also included. Therefore, residual confounding may still be present in those studies where there is a large age difference between patients with AE and controls. Some studies may have included patients with AE, which may have concomitant features, such as myoclonic jerks or atypical features. Also, some of the included studies focused on multiple epilepsy groups and therefore the findings of sub-analyses in patients with AE were often underpowered. Furthermore, some studies were relatively small in sample size and may be subject to a small-study effect. Importantly, multiple comparisons may have yielded false-positive findings (type 1 errors) in the included studies. Furthermore, the Flynn effect, which is a tendency of IQ to rise by ~3 points per decade on average, may have a small but significant bearing on our results (Trahan, Stuebing, Fletcher, & Hiscock, 2014). Unfortunately, inconsistent reporting of neuropsychological test results across studies hindered comparison between studies and pooling of results. Granting that cognitive domains may overlap, assigning neuropsychological tests to a specific cognitive domain may lead to an oversimplified view.

### Future Work

Future work should focus on large (multi-center) long-term observational studies to assess cognitive development and to identify children with AE most at risk for neuropsychological co-morbidity. Importantly, this may clarify whether repeated neuropsychological tests could be helpful and how cognitive development differs from healthy peers. Emphasis should be made to characterize children with AE according to semiology, EEG characteristics, and epilepsy syndrome. Especially separate reporting in JAE is lacking. Early identification of children with concomitant cognitive dysfunctions may help health care workers to initiate additional supportive programs, as well as communicate vulnerabilities and discuss teaching strategies with their school. A broad neuropsychological test battery, focusing on executive functioning, attention and verbal abilities should be used. Ideally, this should ideally be done before the start of anti-epileptic drugs and repeated at least once to track cognitive development. Given that cognitive dysfunctions may be case-specific, personalized supportive programs may be most useful, which may be discussed in a multidisciplinary team, consisting of at least a neurologist, neuropsychologists and a school expert.

## Electronic supplementary material

ESM 1 (DOCX 13 kb)

ESM 2 (PNG 2263 kb)

High resolution image (TIF 120216 kb)

ESM 3 (DOCX 29 kb)

ESM 4 (DOCX 18 kb)

ESM 5 (DOCX 15 kb)
