# Correction to: Towards a Better Understanding of Cognitive Deficits in Absence Epilepsy: a Systematic Review and Meta-Analysis

**DOI:** 10.1007/s11065-019-09425-4

**Published:** 2019-12-20

**Authors:** Eric L. A. Fonseca Wald, Jos G. M. Hendriksen, Gerald S. Drenthen, Sander M. J. V. Kuijk, Albert P. Aldenkamp, Johan S. H. Vles, R. Jeroen Vermeulen, Mariette H. J. A. Debeij van Hall, Sylvia Klinkenberg

**Affiliations:** 1 Department of Neurology, Maastricht University Medical Center+, 6202 AZ Maastricht, The Netherlands; 2 Epilepsy Center Kempenhaeghe, Heeze, The Netherlands; 3 School for Mental Health and Neuroscience, Maastricht University, Maastricht, The Netherlands; 4 Department of Radiology and Nuclear Medicine, Maastricht University Medical Center+, Maastricht, The Netherlands; 5 Department of KEMTA, Maastricht University Medical Center+, Maastricht, The Netherlands; 6 Department of Electrical Engineering, Eindhoven University of Technology, Eindhoven, The Netherlands

**Correction to: Neuropsychology Review**

10.1007/s11065-019-09419-2

Due to an error during the editorial phase, a correction regarding Fig. 2 is added to the original article: “Towards a Better Understanding of Cognitive Deficits in Absence Epilepsy: a Systematic Review and Meta-Analysis”. Please see below the corrected Fig. 2.

**Fig. 2:**
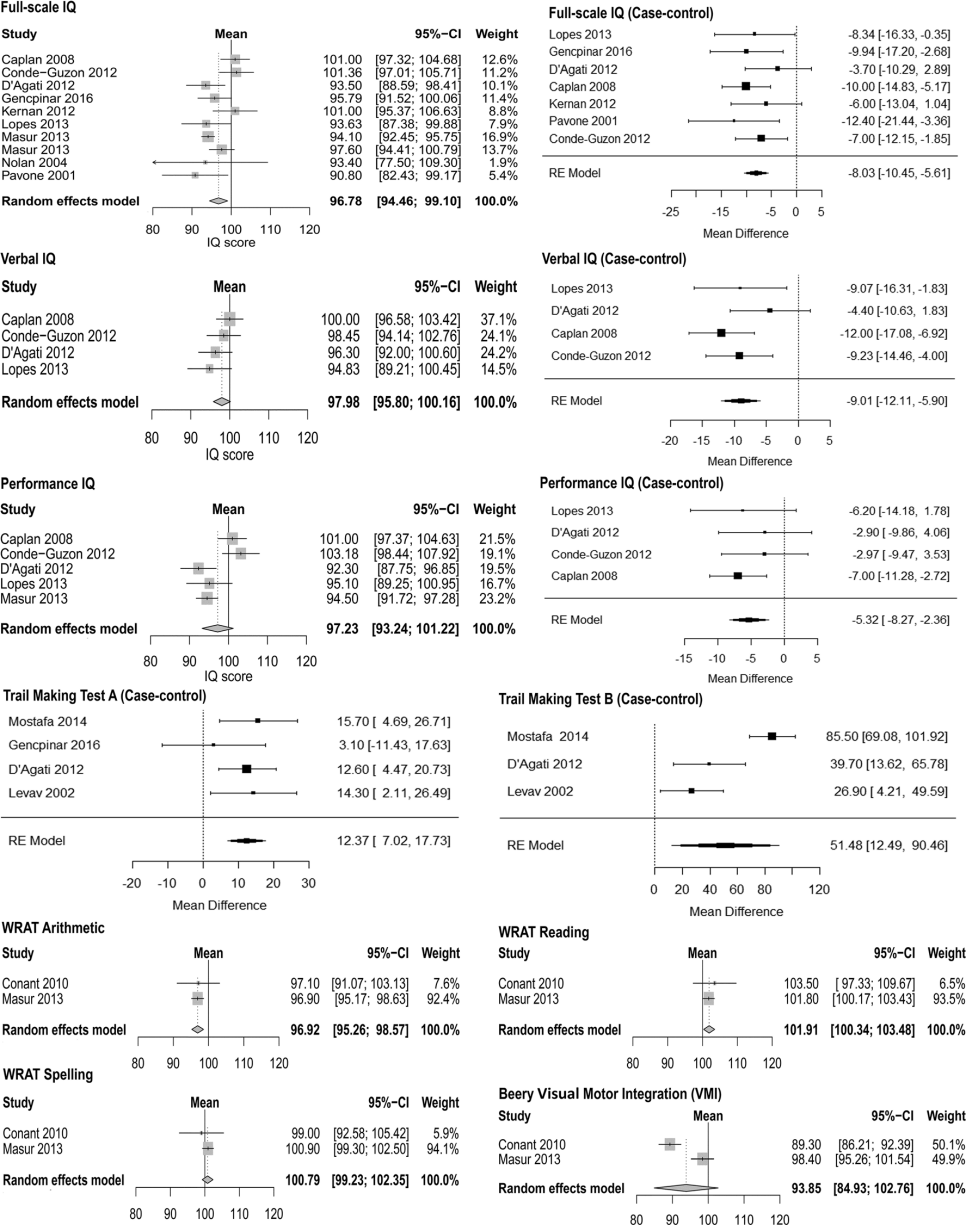
Forest plots of the single-arm meta-analyses (weighted average) and meta-analyses of the mean difference (difference in performance in case-control studies). For each study the mean is represented by a square (size is proportional to the study’s weight) and the 95% confidence interval (CI) is represented by a horizontal line. The overall weighted mean is represented by a diamond shape

