# Supplementary material for: Towards a Better Understanding of Cognitive Deficits in Absence Epilepsy: a Systematic Review and Meta-Analysis
Source: Neuropsychol Rev. 2019 Nov 27;29(4):421–49. doi: 10.1007/s11065-019-09419-2 (PMC6892766; doi:10.1007/s11065-019-09419-2)
Supplement: Supplementary file 1 — (DOCX 13 kb) [file 11065_2019_9419_MOESM1_ESM.docx]

**Exemplary search in Pubmed**

((((((((((("epilepsy, absence"[MeSH]) OR "absence epilepsy") OR "absence seizure") OR "absence seizures") OR "absence epilepsies") OR "pykno-epilepsy") OR "juvenile absence") OR "childhood absence") OR "petit mal") OR "pyknolepsy")) AND (((((((((((((((((((((((prevalence) OR long-term) OR case-control) OR retrospective) OR modifier*) OR risk) OR prognos*) OR "prognosis"[MeSH]) OR predict*) OR "Epidemiologic studies"[MeSH]) OR cohort) OR longitudinal) OR “follow up") OR "followup") OR "follow-up") OR course) OR incidence[TW]) OR "incidence"[MeSH]) OR protect*) OR "epidemiologic factors"[MeSH]) OR "clinical study"[Publication Type]) OR “clinical study”) OR "trial")
