# Supplementary material for: Towards a Better Understanding of Cognitive Deficits in Absence Epilepsy: a Systematic Review and Meta-Analysis
Source: Neuropsychol Rev. 2019 Nov 27;29(4):421–49. doi: 10.1007/s11065-019-09419-2 (PMC6892766; doi:10.1007/s11065-019-09419-2)
Supplement: Supplementary file 5 — (DOCX 18 kb) [file 11065_2019_9419_MOESM4_ESM.docx]

| **Cognitive (sub)domain** | **Reference** | **Test-version** | **Subtest/score** | **N in analysis** | **Results Absence Epilepsy Mean** (**SD)** | **Results Control group Mean** (**SD)** | **P-value or effect-size** |
| --- | --- | --- | --- | --- | --- | --- | --- |
| **Wechsler Intelligence Tests (subtest scores)** | Lopes 2013 | WISCI-III | Information | 30 | 8.70 (2.74) | 11.50 (3.10) | ≤ 0.001* |
|  | Henkin 2005 | WISC-R |  | 12 | 10.4 (1.7) | 11.7 (1.4) | NS |
|  | Lopes 2013 | WISCI-III | Vocabulary | 30 | 10.20 (3.04) | 10.83 (2.63) | NS |
|  | Henkin 2005 | WISC-R |  | 12 | 10.1 (1.9) | 11.05 (1.2) | NS |
|  | Lopes 2013 | WISCI-III | Digit Span | 30 | 7.93 (2.39) | 10.79 (2.96) | ≤ 0.001* |
|  | Kernan 2012 | WISC-III | Digit Span Forward | 31 | 28 (12) | 39 (17) | NS |
|  |  |  | Digit Span Backward |  | 18 (13) | 23 (13) | NS |
|  | D'Agati 2012 | WISC-III | Digit Span Forward | 15 | 4.8 (1.0) | 5.7 (0.8) | 0.055 |
|  |  | WISC-III | Digit Span Backward |  | 3.7 (0.8) | 4.3 (0.6) | 0.129 |
|  | Vanasse 2005 | WISC-III | Digit Span | 10 | 8.4 | 10.6 | NS |
|  | Henkin 2005 | WISC-R |  | 12 | 10.8 (3.8) | 13.7 (4) | < 0.05* |
|  | Levav 2002 | WPPSI-R/WISC-III/WAIS-R |  | 24 | 11.2 (3.9) | 17.5 (4.0) | NA |
|  | Lopes 2013 | WISC-III | Picture Completion | 30 | 9.90 (2.90) | 10.23 (2.96) | NS |
|  | Henkin 2005 | WISC-R |  | 12 | 10.6 (1.5) | 11.6 (1.6) | NS |
|  | Levav 2002 | WPPSI-R/WISC-III/WAIS-R |  | 10 | 15.8 (5.6) Raw score | 19.7 (4.5) Raw score | NA |
|  | Lopes 2013 | WISC-III | Coding | 30 | 8.87 (2.84) | 10.47 (2.69) | NS |
|  | Henkin 2005 | WISC-R |  | 12 | 10.5 (3.2) | 13.7 (3.2) | ≤ 0.01* |
|  | Levav 2002 | WPPSI-R/WISC-III/WAIS-R | Digit Symbol/Coding | 24 | 44.3 (13.9)Raw score | 53.7 (16.9)Raw score | NA |
|  | Lopes 2013 | WISC-III | Block Design | 30 | 9.77 (2.62) | 10.40 (3.78) | NS |
|  | Henkin 2005 | WISC-R |  | 12 | 10.7 (3.1) | 13 (2.2) | NS |
|  | Mostafa 2014 | WISC/WAIS |  | 10 | 7.5 (1.2) | 9.6 (1.4) | NA |
|  | Levav 2002 | WPPSI-R/WISC-III/WAIS-R |  | 10 | 35.9 (15.1) Raw score | 42.2 (13.2)Raw score | NA |
|  | Mostafa 2014 | WISC/WAIS | Similarities | 10 | 9.7 (1.5) | 11 (2.2) | NA |
|  | Levav 2002 | WPPSI-R/WISC-III/WAIS-R | Arithmetic | 24 | 12.5 (3.9) Raw score | 18.0 (5.1)Raw score | NA |
| **Sensory reaction time** | Henkin 2003 | Auditory stimuli | Tonal (Reaction time ms) | 12 | 467.4 (37.5) | 437.3 (18.8) | NS |
|  |  |  | Tonal (Accuracy %) |  | 97.3 (1.8) | 97.5 (1.1) | NS |
|  |  |  | Phonetic “easy” (Reaction time ms) |  | 754.5 (33.5) | 716.3 (21.1) | NS |
|  |  |  | Phonetic “easy” (Accuracy %) |  | 96.8 (1.1) | 99.1 (0.2) | NS |
|  |  |  | Phonetic “difficult” (Reaction time ms) |  | 725.1 (25.1) | 704.6 (18.1) | NS |
|  |  |  | Phonetic “difficult” (Accuracy %) |  | 97.4 (1.1) | 99 (0.2) | NS |
|  |  |  | Semantic (Reaction time ms) |  | 835 (29.1) | 835.2 (24.1) | NS |
|  |  |  | Semantic (Accuracy %) |  | 94.6 (2.4) | 98.2 (0.6) | NS |

Supplementary Table 1: Neuropsychological test results of the included studies per cognitive (sub)domain

^* Significant
N, number of patients; WISC, Wechsler Intelligence Scale for Children; WPPSI, Wechsler Preschool and Primary Scale of Intelligence^
