# Supplementary material for: Towards a Better Understanding of Cognitive Deficits in Absence Epilepsy: a Systematic Review and Meta-Analysis
Source: Neuropsychol Rev. 2019 Nov 27;29(4):421–49. doi: 10.1007/s11065-019-09419-2 (PMC6892766; doi:10.1007/s11065-019-09419-2)
Supplement: Supplementary file 6 — (DOCX 15 kb) [file 11065_2019_9419_MOESM5_ESM.docx]

**Newcastle-Ottawa Quality Assessment Form for Cohort Studies**
Note: A maximum of one star for each numbered item within the Selection and Outcome categories. A maximum of two stars can be given for Comparability.

**Selection**
1) Representativeness of the exposed cohort
Star a) Truly representative
Star b) Somewhat representative
 c) Selected group
 d) No description of the derivation of the cohort

2) Selection of the non-exposed cohort
Star a) Drawn from the same community as the exposed cohort
 b) Drawn from a different source
 c) No description of the derivation of the non-exposed cohort

3) Ascertainment of exposure
Star a) Secure record
 b) Inadequately described

4) Demonstration that outcome of interest was not present at start of study
Star a) Yes (one star)
 b) No

**Comparability (maximum of two stars)**
1) Comparability of cohorts on the basis of the design or analysis controlled for confounders
Star a) The study controls for age and/or sex
Star b) Study controls for other factors (list) _________________________________
 c) Cohorts are not comparable on the basis of the design or analysis controlled for confounders
 **Outcome**
1) Assessment of outcome
Star a) Independent blind assessment
Star b) Validated test
 c) Inadequate description of non-validated test and/or no blind assessment in non-validated tests

2) Sample size
Star a) Justified and satisfactory
 b) Small

3) Statistical analyses
Star a) Appropriate statistical analysis, reporting of all planned analyses
Star b) Only minor problems in statistical analysis
 c) Inappropriate statistical analysis, selective reporting
